# A Deep Learning-Based System for Prawn Hepatopancreas Identification Based on Image Classification with Interpretability

**DOI:** 10.3390/foods15162770

**Published:** 2026-08-07

**Authors:** Dawei Sun, Xianhua Xie, Guanghui Yu, Chen Li, Hongbao Ye, Weiping Fang, Chengquan Zhou

**Affiliations:** 1 Institute of Agricultural Equipment, Zhejiang Academy of Agricultural Sciences, Hangzhou 310021, China; dzs0015@zju.edu.cn (D.S.); ghuiy@cau.edu.cn (G.Y.); lichen@zaas.ac.cn (C.L.); yehb@zaas.ac.cn (H.Y.); 2 Key Laboratory of Agricultural Equipment for Hilly and Mountainous Areas in Southeastern China (Co-Construction by Ministry and Province), Ministry of Agriculture and Rural Affairs, Hangzhou 310021, China; 3 Zhoushan Municipal Bureau of Agriculture and Rural Affairs, Zhoushan 316299, China; dongliangahu@163.com; 4 Agricultural and Rural Bureau of Changxing County, Huzhou 313100, China

**Keywords:** convolutional neural networks, prawn hepatopancreas, image classification, Grad-CAM, explainable artificial intelligence, food quality assessment

## Abstract

Accurate identification of the hepatopancreas is essential for prawn quality assessment and automated seafood processing. This study presents an explainable deep learning framework for the binary classification of prawn images into “with hepatopancreas” and “no hepatopancreas” categories. A custom convolutional neural network (CNN) was developed using a dataset of 252 annotated images. To improve feature extraction from the limited dataset, an image preprocessing pipeline incorporating automatic contour-based cropping, contrast enhancement, and data augmentation was employed. The proposed model achieved a test accuracy of 97.37% and a calibrated full-dataset accuracy of 97.22%. Five-fold cross-validation yielded a mean accuracy of 96.83% ± 1.94%, indicating stable performance across different data partitions. Receiver operating characteristic analysis demonstrated satisfactory discriminative ability with AUC > 0.99. Gradient-weighted Class Activation Mapping (Grad-CAM) showed that the model primarily focused on biologically relevant hepatopancreas regions, improving the interpretability of the classification results. A graphical user interface was developed to enable rapid image analysis with visual feedback. Although further validation using larger and more diverse datasets is required, the proposed framework demonstrates the potential of explainable deep learning for automated prawn quality assessment and provides a practical foundation for intelligent seafood inspection applications.

## 1. Introduction

The giant freshwater prawn *Macrobrachium rosenbergii* is a major species for the aquaculture industry across tropical and subtropical regions, with production volumes expanding steadily in Southeast Asia, South Asia, and parts of the Americas [1]. The commercial value of prawn is heavily influenced by the condition of its hepatopancreas, a large organ situated in the cephalothorax that accumulates lipids, carotenoids, and essential nutrients during growth [2,3]. A highly developed hepatopancreas is prized as a delicacy in many Asian culinary traditions. Specifically, in the Chinese culinary context, the hepatopancreas is commonly referred to as “Gao”, and its presence and condition are key quality indicators in commercial grading. Furthermore, the hepatopancreas also plays a critical role in reproductive processes by providing essential nutrients, making it a key indicator of physiological health and overall product quality [4,5]. Therefore, a well-developed, firm, and vividly colored hepatopancreas is associated with superior flavor, higher nutritional content, and optimal quality, resulting in premium market prices.

The assessment of prawn quality is important in the aquaculture and seafood industries, as it influences both commercial value and consumer safety. Traditional methods for evaluating aquatic products often rely on manual visual inspection and weight [5]. The procedure is time-consuming, labor-intensive, and susceptible to human error, making it a processing bottleneck in high-throughput grading lines. Furthermore, the complex quality trait evaluation, such as hepatopancreas presence, is difficult to visualize, because the non-destructive evaluation of internal tissue color in live crustaceans is inherently limited by the opacity of the exoskeleton [6,7]. Moreover, human evaluation is subjective, with interobserver agreement frequently falling below acceptable thresholds for consistent commercial grading [8]. Consequently, subtle variations in hepatopancreas color and texture may be missed under variable lighting conditions or when the exoskeleton exhibits pigmentation anomalies. These challenges underscore the need for automated, objective, and rapid assessment methodologies.

In recent years, computer vision and deep learning have emerged as transformative technologies for quality assessment across diverse domains, including food quality and safety evaluation. Convolutional neural networks (CNNs), in particular, excel at extracting hierarchical visual features from raw pixel data and progressing from simple edges and textures to complex object parts, thereby surpassing traditional machine learning approaches that depend on manually engineered features [9]. This has led to their successful application in various food-related tasks, such as food classification, grading, defect detection, and freshness evaluation. For instance, deep learning models have been employed to classify the freshness of frozen-thawed shrimp using multiple CNN architectures, achieving high accuracy and demonstrating the potential for automated quality control [10]. Similarly, CNNs have been applied to predict ovarian maturation in prawn broodstock using machine learning approaches, further underscoring the growing role of artificial intelligence in aquaculture management [11]. One recent study has also specifically addressed hepatopancreatic health monitoring using CNNs [12]. These advances demonstrate the feasibility of using deep learning for hepatopancreas-related assessment tasks.

Despite these advances, several challenges remain. A significant bottleneck is the construction of high-quality, annotated datasets, which is often expensive and time-consuming, particularly for specialized tasks like hepatopancreas identification, in which subtle visual cues must be captured. Furthermore, CNN models are often criticized for their “black box” nature, making it difficult to understand the rationale behind their predictions in food quality systems [13]. Gradient-weighted Class Activation Mapping (Grad-CAM) provides a visual explanation by highlighting the input regions that are most influential in the model’s decision, enabling validation that the network attends to biologically relevant anatomy rather than background artifacts [14]. This technique has been successfully applied to fruit ripeness grading [15], fish freshness prediction [16], and edible bird’s nest classification [17], demonstrating its utility in the assessment of complex traits under transparent exoskeleton or layers. To the best of our knowledge, most research on the *M. rosenbergii* hepatopancreas has focused on its physiological functions, nutritional regulation, and disease responses. Currently, no study has addressed the automated identification of hepatopancreas presence in whole specimens using standard RGB imaging and deep learning, despite the fact that hepatopancreas condition is a key quality determinant in commercial prawn grading, particularly in Asian culinary markets where it is prized as a delicacy.

This study aims to develop and evaluate a complete CNN-based system to address the feasibility of automated identification of the presence of prawn hepatopancreas in prawn images. The primary contributions include: (1) the design and implementation of an end-to-end pipeline with a custom preprocessing and data augmentation strategy to overcome the limitations of a modestly sized dataset; (2) a rigorous evaluation of the model’s performance using both validation and test sets; and (3) the integration of Grad-CAM for model interpretability, providing visual evidence that the model’s decisions are based on biologically relevant features. The overall goal is to provide a practical, accurate, and explainable tool for automated prawn quality assessment.

## 2. Materials and Methods

### 2.1. Imaging System and Data Acquisition

**Figure 1:**
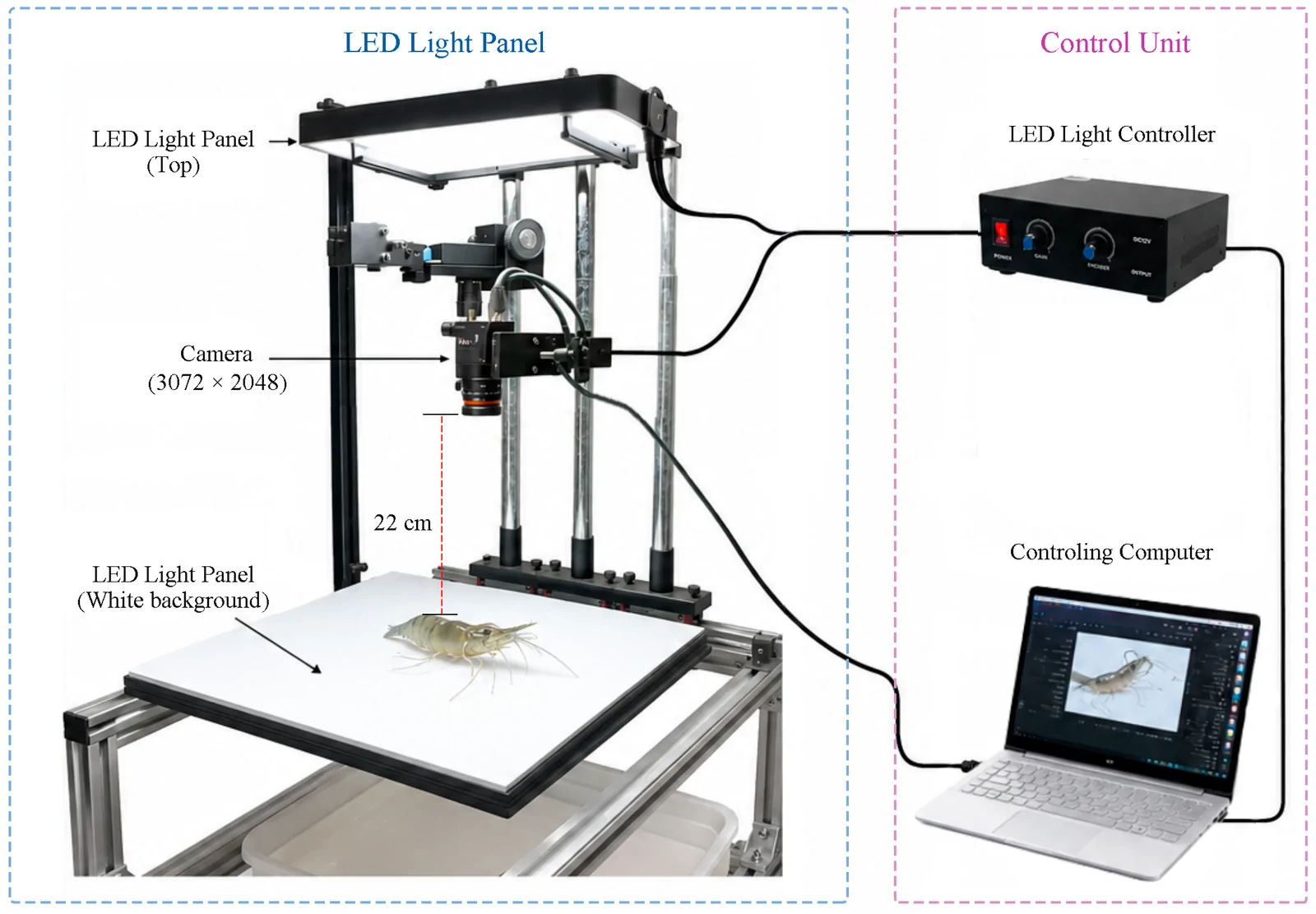
Configuration of the machine vision system for freshwater giant prawn (*Macrobrachium rosenbergii*) image acquisition.

A custom-built machine vision system was employed for high-resolution image acquisition of *M*. *rosenbergii* specimens under controlled illumination conditions (Figure 1). The system comprised an overhead LED illumination panel (JS-FL320320, Huaxing Visual Tech, Dongguan, China), a digital camera (MV-CA060-10GC, Hikrobot, Hangzhou, China), a backlit LED imaging platform (JS-FL400400, Huaxing Visual Tech, Dongguan, China), and a computer-based control unit. The imaging chamber was constructed from an aluminum frame to ensure structural stability and reproducible positioning of all optical components. Uniform top illumination was provided by the square LED panel mounted above the imaging area to minimize shadows and reduce ambient light interference. A second LED panel installed beneath the sample served as a backlit white background, generating high contrast that could facilitate accurate segmentation and enhance the visibility of the hepatopancreas through the translucent exoskeleton. The high-resolution industrial camera (3072 × 2048), equipped with a fixed-focus lens (8 mm, F2.8), was mounted vertically 22 cm above the platform, yielding a spatial resolution of approximately 0.3 mm per pixel, to maintain consistent image quality across all samples. Specimens were placed individually on the illuminated background and imaged from a top-view perspective. Illumination intensity of both LED panels was regulated by a controller to maintain stable lighting throughout the experiment, with the overhead panel set to a range of 800–1200 lux and the backlit panel to 600–1000 lux. The camera was connected to a controlling computer for image acquisition (exposure time at 15 ms), real-time visualization, parameter configuration, and data storage. The combination of uniform top illumination and transmitted backlighting effectively enhanced object-background contrast while suppressing specular reflections from the shrimp exoskeleton, providing high-quality, repeatable images for subsequent deep learning analysis.

### 2.2. Dataset Description and Preprocessing

The dataset consisted of RGB images of market-sized freshwater giant prawns, which were manually annotated according to hepatopancreas visibility. Each image was assigned to one of two categories: “with hepatopancreas” or “without hepatopancreas.” The “No hepatopancreas” category in this study refers to the absence of visible hepatopancreas in RGB images of intact prawns, rather than surgical removal or complete biological degradation. This condition arises from natural variability in hepatopancreas development (e.g., size, lipid accumulation, pigmentation) as well as visual occlusion caused by the opaque exoskeleton, particularly in specimens with thicker shells or darker pigmentation. All specimens were intact market-sized prawns, and the classification task was designed to replicate the visual inspection performed by human graders in commercial processing environments. All prawn specimens (12.31 ± 1.59 g) were purchased from a local market, and all experimental procedures involving prawns were approved by the Institutional Animal Care and Use Committee of the Zhejiang Academy of Agricultural Sciences (Protocol No. 25ZALAS24). The dataset was randomly partitioned into training, validation, and test sets with a ratio of approximately 70%, 15%, and 15%, resulting in 176, 38, and 38 images per set, respectively.

The relatively small dataset reflects the practical difficulty of acquiring manually annotated hepatopancreas images under standardized conditions. A multi-stage preprocessing pipeline was implemented to enhance the quality of the input data and standardize it for the subsequent CNN modeling. An automatic cropping procedure was first applied, which included background segmentation followed by contour detection to identify the region of interest (ROI). The image was then cropped to this bounding box. Then, a series of image enhancement techniques was applied to amplify the discriminative features associated with hepatopancreas identification, including contrast enhancement, saturation adjustment, and sharpening, which collectively served to make the color and texture differences between the hepatopancreas and the surrounding prawn tissues more pronounced.

### 2.3. Data Augmentation

To mitigate the potential risk of overfitting, a comprehensive online data augmentation strategy was employed during the training phase. Unlike offline augmentation, which generates a fixed set of augmented images prior to training, online augmentation applies random transformations to each training sample dynamically at the beginning of each epoch. This approach ensures that the model encounters a different variation in each sample in every epoch, greatly diversifying the training distribution without increasing storage requirements. The augmentation pipeline included random horizontal and vertical flips; rotations within ±20 degrees; affine transformations with random shear and scale; color perturbation adjusting brightness, contrast, saturation, and hue; and random erasing of small rectangular patches. The model was trained for 60 epochs on a training set of 176 samples, resulting in exposure to 176 × 60 = 10560 augmented training views. This substantial increase in effective training data significantly enhanced the model’s ability to generalize to unseen samples.

### 2.4. Model Architecture

**Figure 2:**
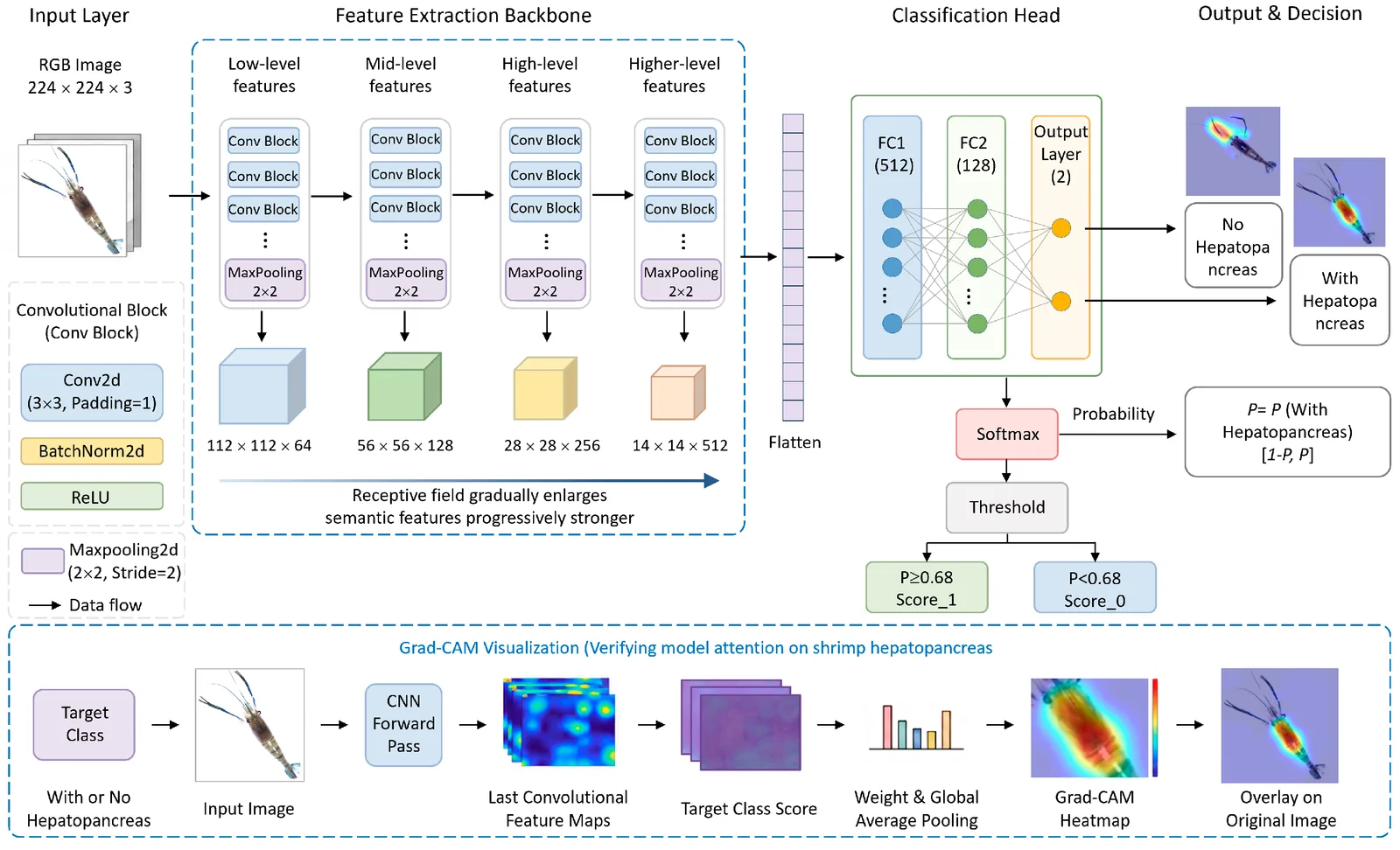
The framework of CNN with interpretability.

The classification model was built using a custom CNN implemented with the PyTorch 2.7.1 deep learning framework. The network takes 224 × 224 RGB images as input. The architecture comprises a sequence of convolutional layers, each followed by batch normalization and Rectified linear unit (ReLU) activation functions. Max-pooling layers are interspersed to progressively reduce the spatial dimensions of the feature maps while retaining salient information. This design allows the network to extract increasingly complex features from the raw pixels: initial layers capture low-level features like edges and colors, while deeper layers combine these to form high-level representations corresponding to shapes and textures indicative of hepatopancreas presence or absence. The architecture of the network is illustrated in Figure 2.

The final feature maps are flattened and passed to a series of fully connected layers, culminating in a binary output layer. This output layer uses the softmax activation function to produce a probability distribution over the two classes: “No hepatopancreas” and “With hepatopancreas”. The classification decision was determined based on the predicted probability of the “With hepatopancreas” class. Threshold calibration was performed using the validation dataset by evaluating candidate thresholds according to sensitivity, specificity, Youden’s J statistic, F1-score, and validation accuracy (Supplementary Table S2). A threshold of 0.68 was selected because it achieved the highest validation accuracy (92.11%) and Youden’s J statistic (0.85), while maintaining a high F1-score (0.96). An image was classified as having hepatopancreas if its “with hepatopancreas” probability was ≥0.68; otherwise, it was classified as “No hepatopancreas.” The independent test set was subsequently evaluated using this fixed threshold without further optimization.

The model was trained using the Adam optimizer with a standard cross-entropy loss function, suitable for binary classification. The initial learning rate was set to 0.001 with a cosine annealing scheduler, and a batch size of 16 was employed. To prevent overfitting, an early stopping strategy with a patience of 16 epochs was applied, and model weights were saved based on the best validation performance. Training was conducted on an NVIDIA RTX 3080 GPU (10 GB VRAM) with CUDA 11.8 and cuDNN 8.9, with deterministic cuDNN operations enabled to ensure reproducibility. The training process was monitored by tracking training and validation accuracy and loss curves to diagnose potential overfitting and confirm convergence. Key training hyperparameters and model configurations are listed in Supplementary Table S1.

### 2.5. Interpretability via Grad-CAM

To enhance the interpretability of the CNN’s decision-making process, Grad-CAM was implemented (Figure 2). Grad-CAM uses the gradient information from the final convolutional layer to produce a coarse localization map. This map highlights the regions in the input image that are most influential for the model’s prediction for a specific class. For a given input image and target class, the forward pass and backpropagation are performed to compute the gradients of the class score with respect to the feature maps of the final convolutional layer. These gradients are then global-average-pooled to obtain importance weights for each feature map. A weighted combination of these feature maps followed by a ReLU activation produces the heatmap that highlights the relevant image regions. The resulting heatmap is then upsampled to the original image size and overlaid, providing a visual explanation of the model’s focus. In this study, Grad-CAM heatmaps were generated for both correctly and incorrectly classified samples to validate that the activation maps primarily localized to the hepatopancreas region when it was present and to diagnose potential sources of error.

### 2.6. Evaluation Metrics

Model performance was evaluated by calculating multiple complementary metrics, including accuracy, precision, recall, specificity, F1-score, and Mathew’s correlation coefficient (MCC), using the following equations. Confusion matrices were generated for the test set and the full dataset to provide a detailed breakdown of true positives (TP), true negatives (TN), false positives (FP), and false negatives (FN), allowing the overall understanding of the model’s strengths and weaknesses. To quantify the uncertainty associated with the limited test set, 95% confidence intervals for the primary performance metrics were computed using the Wilson score method, which provides more reliable interval estimates than the normal approximation for small sample sizes.

(1) Accuracy = (TP + TN)/(TP + TN + FP + FN)

(2) Precision = TP/(TP + FP)

(3) Recall (Sensitivity) = TP/(TP + FN)

(4) Specificity = TN/(TN + FP)

(5) F1-Value = 2 × TP/(2TP + FP + FN)

(6) Matthews Correlation Coefficient (MCC) = (TP × TN − FP × FN)/√ [(TP + FP) (TP + FN) (TN + FP) (TN + FN)]

To further evaluate model robustness and generalizability, stratified five-fold cross-validation was conducted using the complete dataset to preserve the class distribution in each fold. The dataset was randomly divided into five approximately equal subsets, with four folds used for training and the remaining fold used for validation. This procedure was repeated five times so that each fold served as the validation set once. The mean classification accuracy and standard deviation across the five validation folds were subsequently calculated to assess the stability of the proposed model under different data partitions. In addition, considering the limited dataset size, the receiver operating characteristic (ROC) curve and the corresponding area under the curve (AUC) were used to provide a comprehensive evaluation of classification performance.

## 3. Results

### 3.1. Training Performance

**Figure 3:**
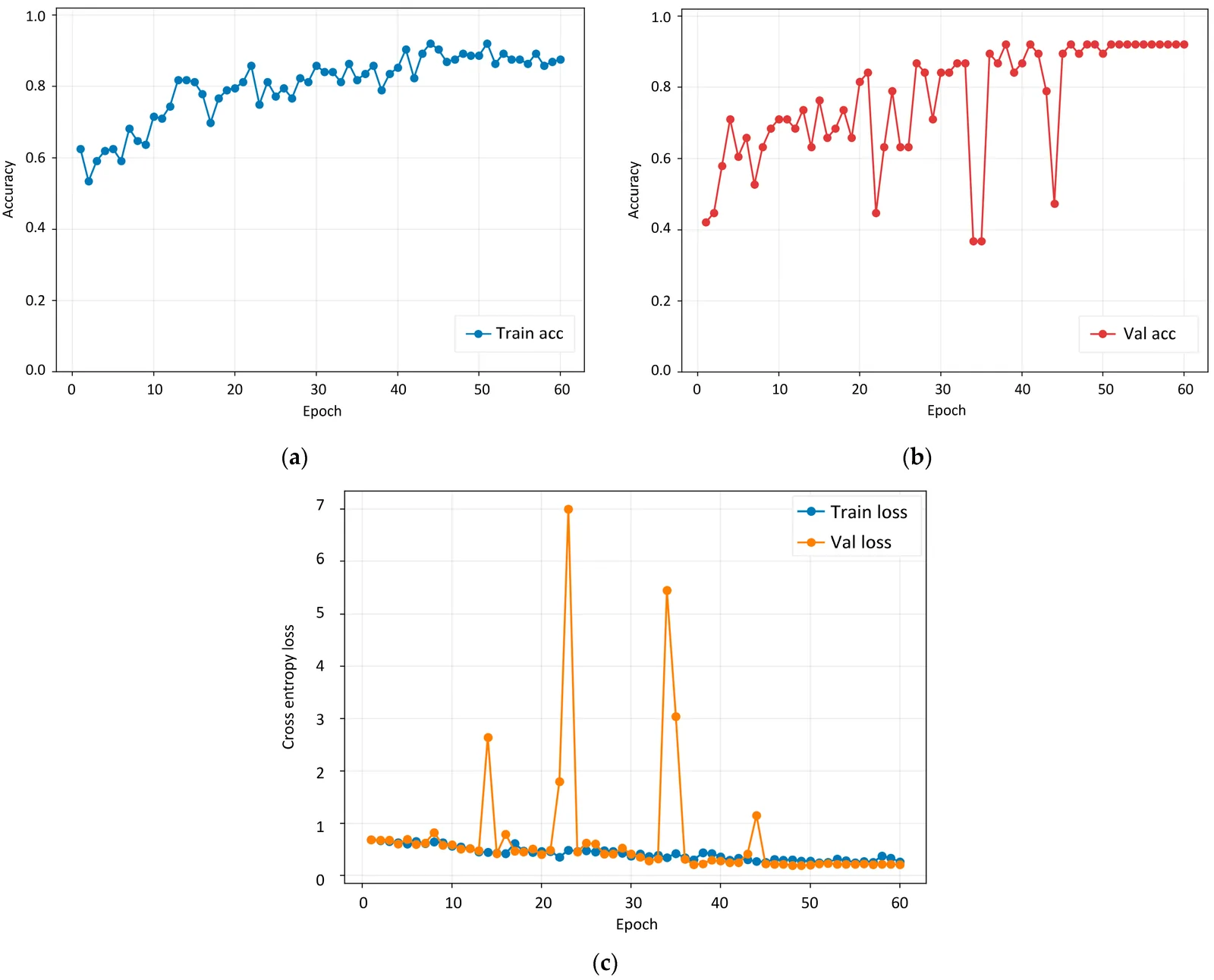
The training and Validation Dynamics. (**a**) The training accuracy; (**b**) the validation accuracy curve; (**c**) the cross-entropy loss curve.

The final dataset included 252 annotated images, comprising 157 images with visible hepatopancreas and 95 images without visible hepatopancreas. The training process was monitored through accuracy and loss curves across epochs (Figure 3). Training accuracy increased steadily from approximately 65% at initialization to 97.72% by the final epochs, indicating effective fitting to the training distribution (Figure 3a). The validation accuracy curve exhibited a peak at epoch 49 with 92.11% accuracy, followed by a gradual decline in subsequent epochs, supporting the checkpoint preservation strategy (Figure 3b). The validation loss reached its minimum value of 0.1938 at the same epoch, providing convergent evidence for the optimality of the epoch 49 weights (Figure 3c). The training and validation accuracy curves, as well as the loss curves, indicated a stable training process without significant overfitting. The decision to save the best validation model rather than the final epoch’s model ensured that the most generalizable weight configuration was retained for evaluation.

### 3.2. Analyzation of Prediction Performance

A comprehensive analysis was conducted to summarize the model’s predictions on the full dataset. The probability distribution plot showed the distribution of the model’s confidence scores for the “with hepatopancreas” class across all samples, revealing a clear separation between the two classes, with most positive samples receiving high confidence scores and negative samples receiving low scores. The confusion matrix of the full dataset provided a visualization of the true and predicted classes, confirming the high overall accuracy. Finally, the misclassified sample plot presented the probability scores for the few errors, offering insights into the “hard” cases in which the model’s decision was close to the threshold. Such visual tools are valuable to allow users to quickly assess the system’s reliability.

#### 3.2.1. Probability Distribution and Confidences

**Figure 4:**
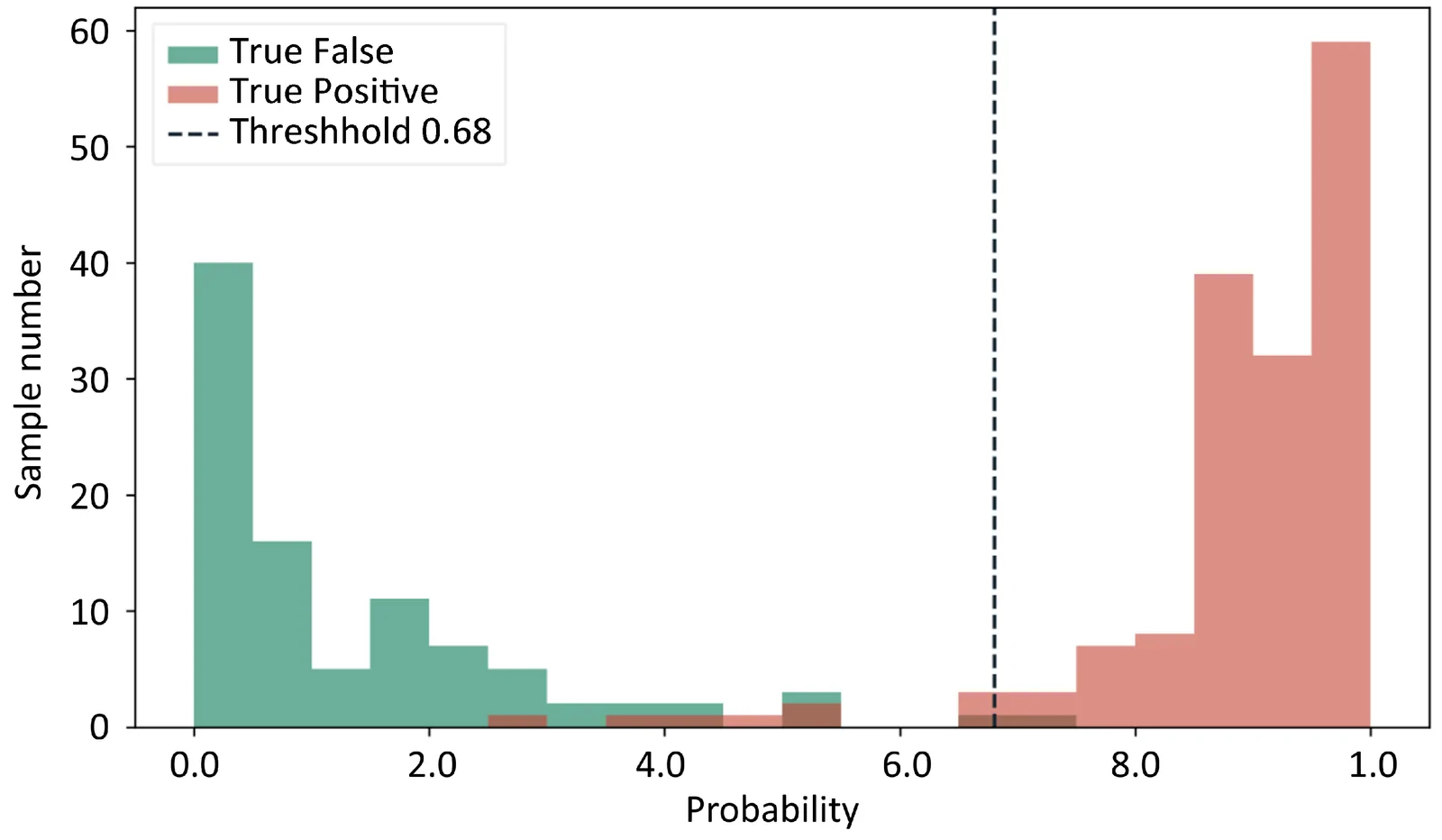
The probability Distribution Map.

The decision threshold was optimized using the validation dataset. As shown in Supplementary Table S2, the conventional threshold of 0.50 resulted in high sensitivity but lower specificity, increasing the risk of false-positive predictions. The threshold of 0.68 achieved the best balance between sensitivity and specificity, with the highest validation accuracy (92.11%) and Youden’s J statistic (0.85). Therefore, this threshold was selected for subsequent evaluation. The predicted probabilities for the positive class showed strong separation from the negative class (Figure 4). Positive samples exhibited probability values predominantly above 0.80, concentrated near the upper end of the distribution. Negative samples showed probability values mostly below 0.40, clustered near the lower end. This bimodal distribution indicated that the model had learned highly discriminative features for the majority of samples. The small overlap region between the two distributions, centered approximately in the 0.50 to 0.70 range, corresponded to the minority of misclassified samples and represented inherently ambiguous cases in which hepatopancreas condition was visually intermediate or confounded by other anatomical structures.

#### 3.2.2. Classification Accuracy and Confusion Matrix Analysis

**Figure 5:**
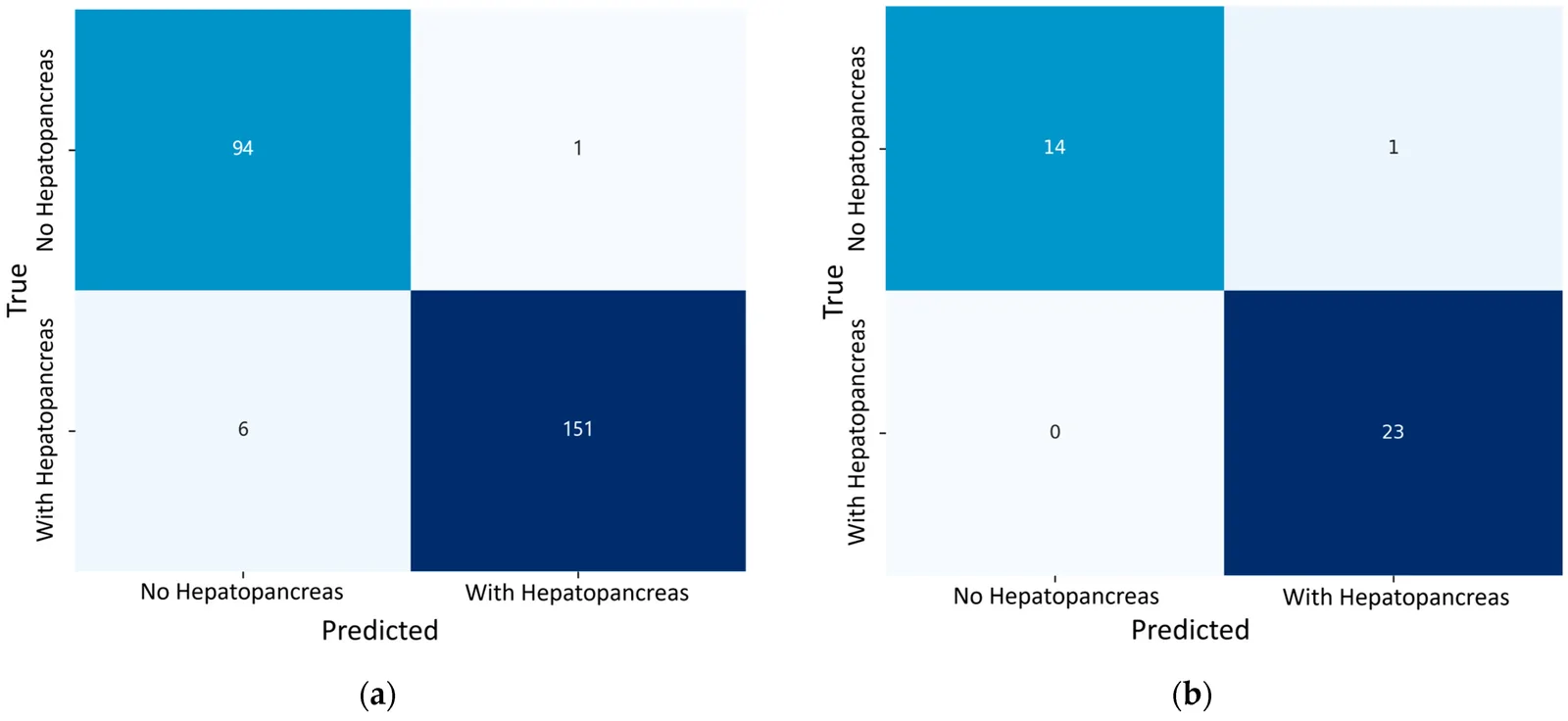
Confusion matrix: (**a**) Full dataset. (**b**) Test dataset.

Application of the calibrated threshold at 0.68 maintained validation accuracy at 92.11% and test accuracy at 97.37%, confirming that the calibration did not degrade performance. Evaluation across the complete annotated dataset of 252 images yielded a calibrated accuracy of 97.22%, corresponding to 245 correct classifications and 7 errors (Figure 5a). The model’s classification performance was evaluated on the independent test set (Figure 5b). It achieved a satisfactory test set accuracy of 97.37%, demonstrating strong generalization. Analysis of the test set confusion matrix revealed that among the 15 “No hepatopancreas” samples, 14 were correctly classified with one false positive error. All 23 “With hepatopancreas” samples were correctly identified. This result demonstrates the high sensitivity and specificity of constructed model. This consistency across splits indicated that the model’s performance generalized uniformly across the entire available data.

This high performance on the full dataset corroborates the test set findings and indicates the model’s capability to generalize well to unseen images. The calibration of the decision threshold proved effective in balancing classification performance for both classes. Table 1 summarizes the key performance metrics.

#### 3.2.3. Comprehensive Performance Metrics

Given the relatively small dataset, additional performance metrics were employed to provide stronger justification of the reliability of the performance, including precision, recall, specificity, F1-score, Matthew’s correlation coefficient, ROC curves, and AUC. On the independent test set, the model achieved satisfactory metrics (Table 2).

Based on the independent test set, the model demonstrated satisfactory classification performance on the test set, achieving an accuracy of 97.37% with 95% CI between 86.51–99.53% and a recall value of 100%. The high precision and specificity further indicate the model’s ability to correctly identify both positive and negative cases, while the Matthews Correlation Coefficient of 0.9458 suggests strong agreement between predictions and ground-truth labels. The relatively wide confidence intervals reflect the limited data size and underscore the need for validation on larger independent datasets to refine performance estimates. Five-fold cross-validation yielded a mean accuracy of 96.83% ± 1.94%, indicating stable performance across different data partitions.

**Figure 6:**
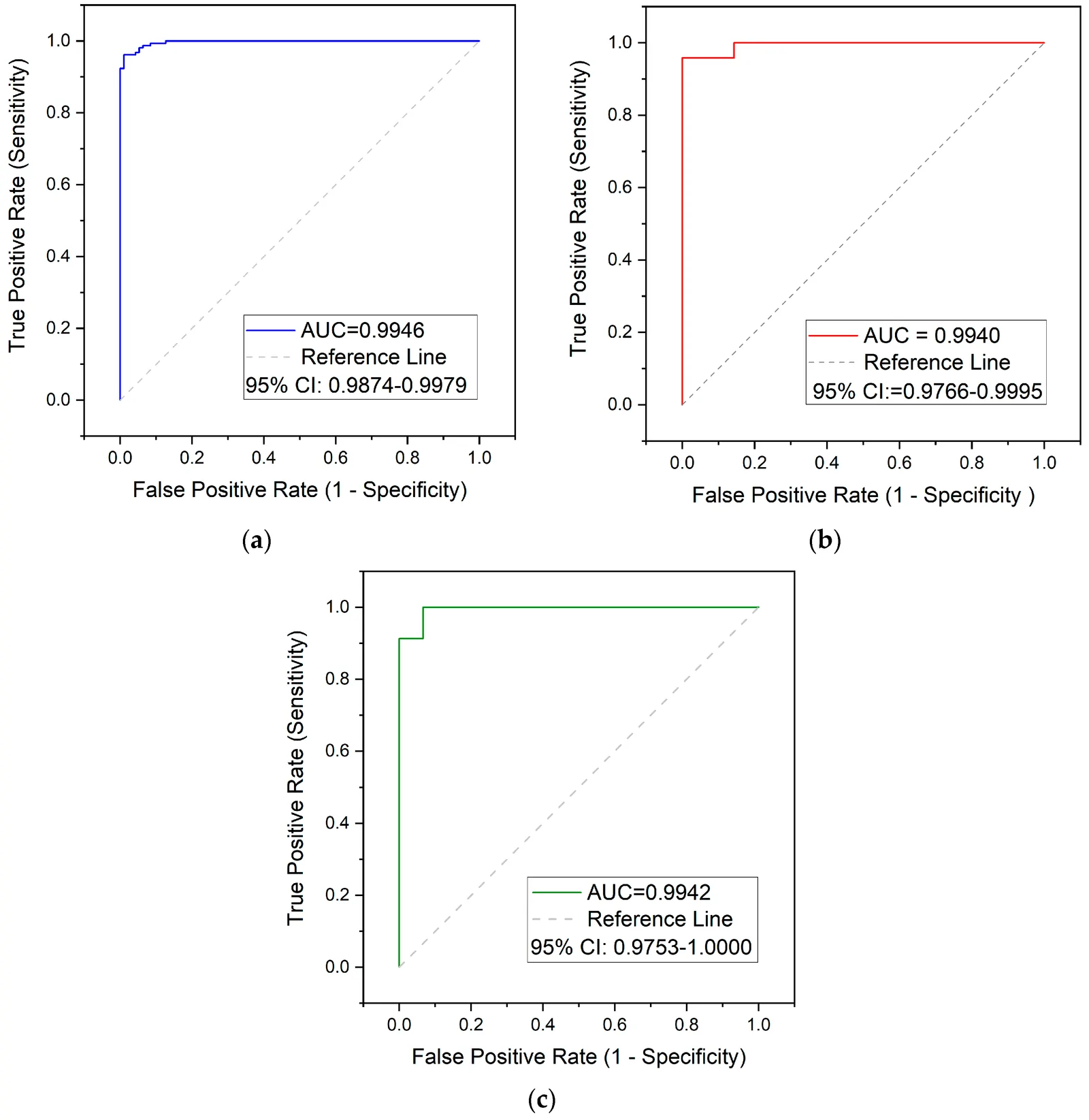
The receiver operating characteristic curve (ROC). (**a**) ROC for the full dataset (n = 252); (**b**) ROC for the validation dataset (n = 38); (**c**) ROC for the test dataset (n = 38).

Figure 6 presents the ROC curves of the proposed CNN for the full dataset, validation set, and independent test set, respectively. In all three datasets, the ROC curves are concentrated near the upper-left corner, indicating excellent discrimination between prawns with and without hepatopancreas. The corresponding AUC values were 0.9946 for the full dataset, 0.9940 for the validation set, and 0.9942 for the test set, demonstrating consistently high classification performance across different data partitions. The close agreement among the three ROC curves suggests that the model maintained stable predictive capability during training and evaluation, with no evidence of substantial performance degradation on unseen data. Overall, these results indicate that the proposed CNN achieved high sensitivity while maintaining a low false-positive rate, supporting its robustness and reliability for automated hepatopancreas classification under the experimental conditions employed in this study.

### 3.3. Interpretability via Model Focus Based on Grad-CAM

**Figure 7:**
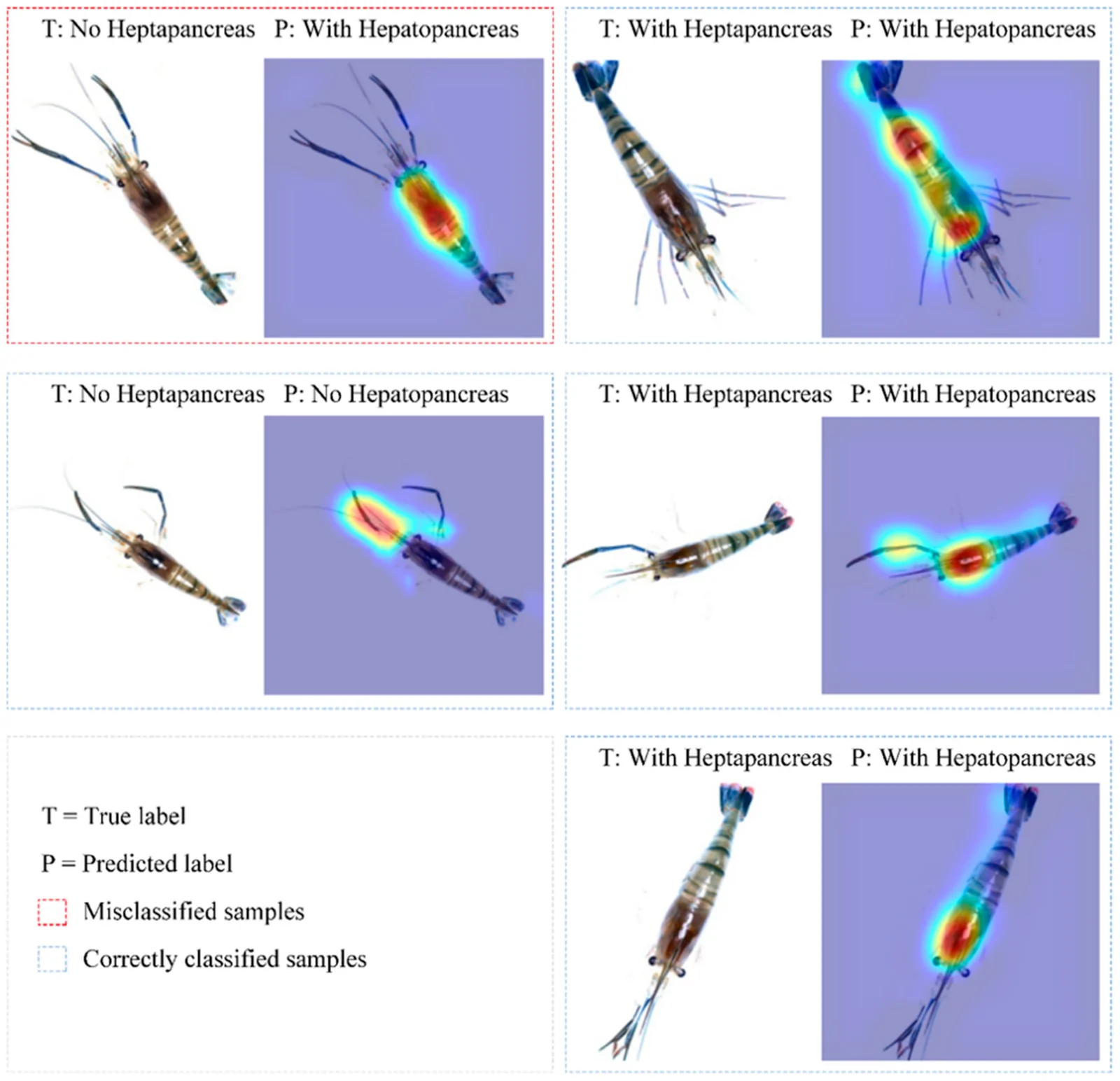
Grad-CAM heat maps of correctly and mis-classified samples for interpretability.

To further investigate the interpretability of the proposed deep learning model and evaluate whether the classification decision was influenced by prawn orientation or spatial arrangement, Grad-CAM was employed to visualize the image regions contributing most strongly to the model predictions (Figure 7). The samples presented in Figure 7 intentionally include variations in prawn orientation and position to assess the robustness of the learned features against moderate geometric variations. Since all images were acquired under a standardized top-view imaging protocol, these variations represent common positional differences that may occur during practical image acquisition rather than uncontrolled acquisition conditions.

For correctly classified samples, the generated heatmaps consistently focused on the cephalothorax region, which corresponds anatomically to the location of the hepatopancreas, despite differences in object orientation and placement. In prawns with visible hepatopancreas, the activation maps exhibited strong responses within the cephalothorax region and occasionally extended into adjacent anterior abdominal segments. In contrast, samples without visible hepatopancreas generally showed weaker and more localized activation patterns. These results indicate that the CNN primarily learned hepatopancreas-associated visual features rather than relying on object orientation, background information, or fixed spatial positioning for classification.

Notably, minimal activation was observed in the antennae, pleopods, and background regions, further demonstrating that the model did not depend on irrelevant image characteristics. The consistency of the highlighted regions across different orientations suggests that the learned representations were associated with biologically meaningful characteristics of the hepatopancreas rather than orientation-specific patterns.

Grad-CAM analysis was also performed on misclassified samples to identify potential sources of prediction error. In several cases, the model’s attention was partially directed to regions exhibiting strong texture variations, shadows, specular reflections, or contrast transitions between the cephalothorax and abdominal segments. Such visual disturbances may have interfered with the extraction of hepatopancreas-related features, leading to incorrect predictions. Additionally, some misclassified individuals possessed relatively small, poorly developed, or partially occluded hepatopancreas tissues, resulting in weak visual cues and increased classification uncertainty. These observations highlight the challenges associated with subtle phenotypic variation and emphasize the importance of incorporating a larger and more diverse dataset that includes difficult samples in future studies.

Overall, the Grad-CAM visualizations indicate that the proposed model predominantly bases its decisions on anatomically meaningful regions associated with the hepatopancreas. The agreement between model attention and biological knowledge enhances the transparency and reliability of the classification framework, providing additional confidence in its application for automated hepatopancreas identification in *M. rosenbergii*.

### 3.4. Comparison with Established Architectures

To provide stronger justification and further strengthen the reliability of the model performance, we conducted a comprehensive benchmark comparison against five established deep learning architectures widely used for image classification, namely ResNet-18, EfficientNet-B0, MobileNetV3, DenseNet-121, and VGG-16. All models were trained and evaluated under identical conditions using the same dataset splits, data augmentation strategy, and training hyperparameters as the proposed CNN. Table 3 compares the classification performance of the proposed CNN with five widely used deep learning architectures.

As shown in Table 3, the proposed CNN achieved a test accuracy of 97.37%, together with the highest recall and F1-score, indicating the effective identification of prawns with hepatopancreas. Although EfficientNet-B0 achieved the same test accuracy (97.37%) and a marginally higher MCC, the proposed CNN attained perfect recall while maintaining competitive precision (95.83%) and specificity (93.33%). In contrast, ResNet-18, MobileNetV3, DenseNet-121, and VGG-16 achieved lower test accuracies (92.11–94.74%) and MCC values (0.8341–0.8976). Overall, the proposed CNN demonstrated classification performance comparable to or better than the benchmark architectures across most evaluation metrics.

The performance of the proposed system is competitive with that of other deep learning applications in the seafood domain. For instance, a study on predicting ovarian maturation in *Penaeus monodon* using a random forest model achieved an accuracy of 88.5% [11], while the CNN + LSTM model for prawn freshness classification reported a 99.07% accuracy rate [10]. The high accuracy of 97.37% achieved in this study with the limited dataset of 252 images underscores the effectiveness of the custom preprocessing and data augmentation strategy. It suggests that with careful data handling and enhancement, deep learning models can achieve satisfactory results even when large-scale datasets are not available, which is a common constraint in specialized applications. Furthermore, the explicit integration of model interpretability via Grad-CAM aligns with recent trends in the field, which emphasize the need for explainability for deep learning systems.

### 3.5. Graphical User Interface Development

**Figure 8:**
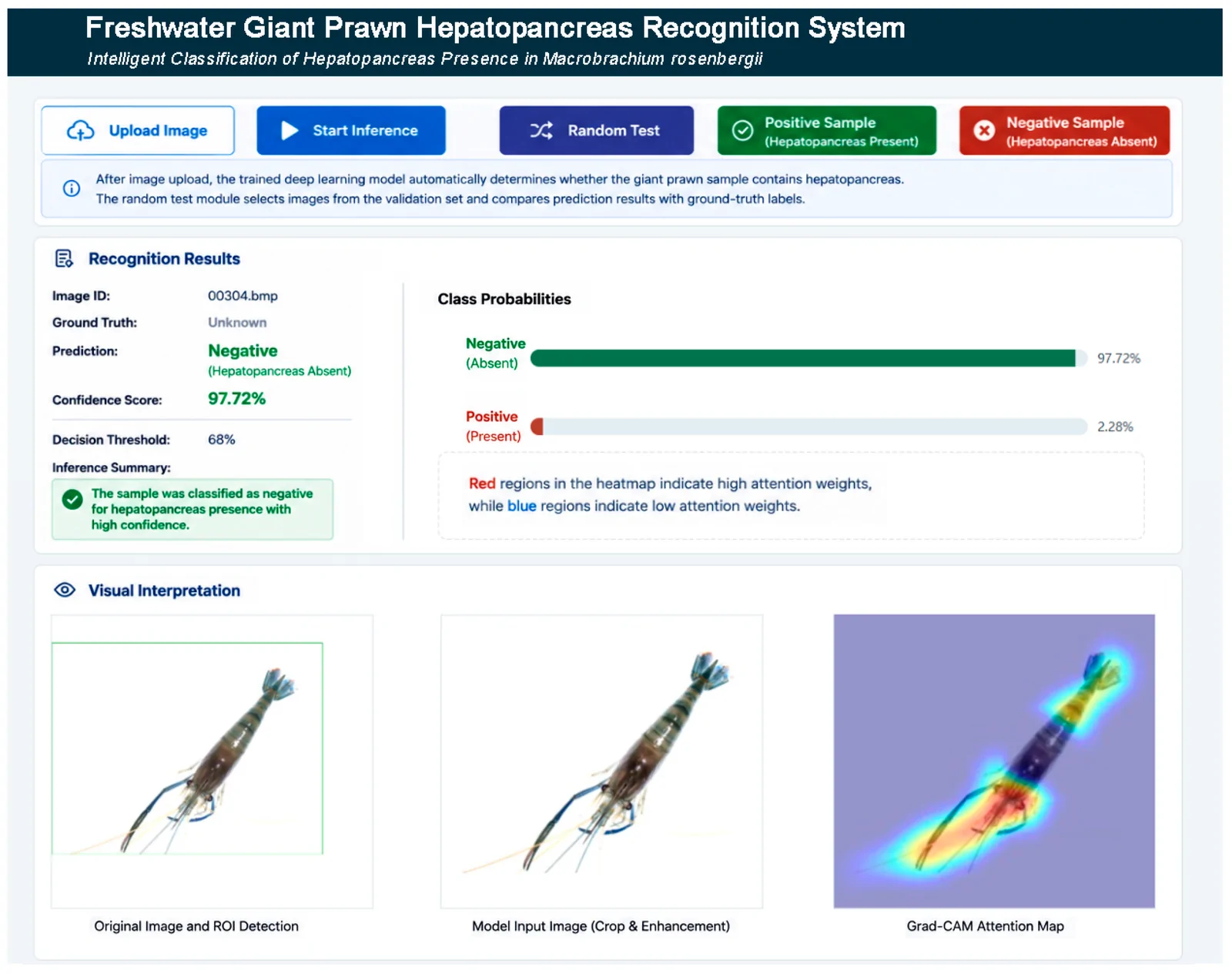
Graphical user interface for freshwater giant prawn hepatopancreas classification.

To facilitate the practical deployment of the developed classification system, a simple GUI was developed (Figure 8). The interface consists of four integrated modules, including image uploading, preprocessing visualization, classification output, and model interpretability. Users can upload images or select random samples for testing, and the preprocessing panel displays both the original image with the detected region of interest and the cropped image used as model input. The prediction panel provides classification results along with confidence scores and class probabilities. To improve model transparency, Grad-CAM visualizations are generated simultaneously, highlighting the image regions that contribute most strongly to the classification decision. Overall, the developed interface provides an efficient and user-friendly platform for practical hepatopancreas inspection while enhancing the interpretability and reliability of model predictions.

## 4. Discussion

### 4.1. Feasibility of High Accuracy with Limited Samples

Small-sample image recognition has attracted considerable attention in computer vision because many real-world applications have limited labeled data. Under these conditions, conventional deep learning models are prone to overfitting and often exhibit poor generalization. To overcome these limitations, data augmentation has been widely adopted to improve recognition performance with limited training data [18]. Overall, the achievement of high classification performance from a modest image dataset could be well justified by the task characteristics, data quality, and methodological rigor. Therefore, it is not uncommon for CNNs to achieve excellent results on a small dataset, with well-defined dataset and justified process [19,20]. The binary classification nature of this study combined with the hepatopancreas’s distinct chromatic and anatomical signature substantially reduces the feature space complexity compared to multiclass or general-purpose recognition problems. Our standardized imaging protocol eliminates environmental confounders, while the aggressive online augmentation effectively expands the sample diversity to a scale comparable to much larger static datasets [21]. Finally, the comprehensive validation metrics validation further provides strong empirical evidence that the results are stable and not attributable to favorable data partitioning. Our findings align with established precedents in food quality and aquaculture imaging, where high-performance deep learning models have been routinely achieved on datasets of comparable size when visual features are well-defined and imaging conditions are controlled [22]. On this basis, these considerations support the viability of the proposed approach using the current dataset for controlled testing. We stress, however, that independent validation on datasets of biological samples from multiple facilities and across different years will be necessary to confirm robustness and generalizability before potential commercial adoption.

Importantly, while online augmentation generates 10,560 training views from 176 unique images, these augmented samples do not introduce new biological information beyond the transformations applied to existing specimens. The ratio of augmented views to unique biological samples is high, and a legitimate concern is that the model may learn to exploit augmentation artifacts rather than generalizable biological features. However, several observations mitigate this concern: first, the Grad-CAM visualizations consistently localized to anatomically relevant regions, indicating that the model’s decisions are based on biologically meaningful features rather than background or texture artifacts. Second, the 5-fold cross-validation results (96.83% ± 1.94%) suggest that the model generalizes across different data partitions of the same underlying population. Third, the consistent performance across the test set (97.37%) indicates that the model has not simply memorized the training samples. Nevertheless, we acknowledge that the true test of generalization will require independent validation on biologically distinct samples from different facilities, seasons, and production batches.

### 4.2. Efficacy of Preprocessing and Augmentation in Small-Sample Learning

The achievement of 97.22% overall accuracy underscores the effectiveness of the preprocessing and augmentation pipeline. Automatic cropping eliminated background-related variability and reduced the input space to diagnostically relevant anatomy. This is particularly important for hepatopancreas assessment, as the organ’s appearance must be evaluated through the exoskeleton. The contrast and saturation enhancements successfully amplified the subtle color differences between intact and degraded hepatopancreatic tissues, which primarily arise from variations in carotenoid and lipid content [23,24]. Previous research has established significant correlations between shell color parameters and hepatopancreas carotenoid content in *M. rosenbergii*, with the a* and b* values of shells showing significant positive correlations with astaxanthin content in the hepatopancreas [2,25]. These findings support the rationale that external visual characteristics can serve as reliable proxies for internal hepatopancreas quality.

The augmentation strategy effectively increased the effective training set size without additional annotation cost. Geometric transformations addressed variability in prawn positioning, while photometric perturbations simulated the range of illumination conditions likely to be encountered in processing plants. Studies have demonstrated that augmented shrimp datasets achieve superior performance compared to non-augmented datasets in quality classification tasks [26]. Previous work in small-sample agricultural vision has shown that such augmentation can improve generalization by 5–15% compared to training without augmentation [27,28,29]. Our results are consistent with these findings and confirm that even with a modest base dataset, carefully designed augmentation enables deep learning to achieve practical accuracy levels.

### 4.3. Training Dynamics and Loss Fluctuation Analysis

Throughout the training process, the cross-entropy loss exhibited a well-behaved overall convergence trend, with both training and validation losses declining steadily toward their respective minima. However, there were occasional large-magnitude loss spikes that rapidly returned to baseline levels in subsequent epochs. Several factors might contribute to these expected fluctuations. The online data augmentation strategy employed during training might have introduced stochastic variations in each training sample at every epoch [30]. These transformations occasionally generated partially occluded or distorted hepatopancreatic features, resulting in temporarily elevated loss values. Another reason might be the mini-batch stochastic gradient descent process, which inherently introduces variability in gradient updates across different batches [31]. Therefore, a difficult sample paired with a challenging batch composition in one epoch may produce higher loss than when paired with favorable samples in another epoch. Finally, the presence of intrinsically ambiguous samples near the decision boundary could contribute to these fluctuations [32]. In the present dataset, some specimens exhibited hepatopancreatic conditions that were visually intermediate. These samples produced higher classification uncertainty, leading to larger variance across epochs. However, these transient fluctuations did not affect the overall convergence, as reflected by the steady decline in training loss and validation accuracy.

This pattern of loss fluctuation is a known characteristic of deep learning optimization on datasets containing heterogeneous sample difficulty [33]. The observed behavior indicates that the model successfully learned discriminative features for the majority of samples while continuing to iteratively adjust its decision boundary for challenging borderline cases, ultimately achieving robust classification performance on both validation and test sets.

### 4.4. Threshold Calibration and Biological Validity for Interpretability

The calibrated decision threshold of 0.68 for the hepatopancreas class has important practical implications. In commercial processing, the cost of incorrectly upgrading a degraded sample to premium grade typically exceeds the cost of downgrading an intact sample, as the former results in a direct quality complaint and potential consumer rejection, while the latter merely reduces the sale price. The elevated threshold thus introduces a conservative bias toward the positive class when the model is uncertain, which aligns with commercial risk preferences.

The Grad-CAM visualizations provide critical validation that the model’s decisions are biologically grounded. Unlike some image classification applications in which models inadvertently learn background or labeling artifacts, the present network consistently localized its attention to the hepatopancreatic region of the cephalothorax. This finding increases confidence that the model will generalize to new imaging conditions, as it relies on the actual biological signal rather than on empirical patterns alone [34]. This approach agrees with the study of explainable AI in shrimp health monitoring, in which visualization techniques have been employed to build trust in automated diagnostic systems [10]. Therefore, the integration of Grad-CAM into the graphical interface further supports operator acceptance, as quality personnel can visually verify the basis of each prediction before accepting or overriding it.

### 4.5. Comparison with Related Work

Most previous studies on the hepatopancreas of *M. rosenbergii* have focused on physiological functions, nutritional regulation, and disease responses, whereas the application of computer vision and deep learning has mainly been limited to histological tissue analysis for disease diagnosis [35,36]. To date, automated identification of hepatopancreas presence in whole prawns using standard RGB imaging has rarely been explored. This study addresses this gap by demonstrating the feasibility of nondestructive hepatopancreas status classification, which is particularly relevant because hepatopancreas condition is an important quality attribute in commercial prawn grading, especially in Asian markets.

The proposed approach also compares favorably with previous deep learning-based applications in shrimp and crustacean quality assessment. CNN-based methods have achieved over 95% accuracy for shrimp hepatopancreatic disease identification using tissue samples [12], while deep learning models have been successfully applied to shrimp color grading and freshness assessment [37,38]. Unlike these approaches, the present study enables high-accuracy classification of hepatopancreas status directly from whole-prawn RGB images without tissue sectioning or specialized imaging equipment, providing potential advantages for rapid and nondestructive quality inspection.

Although the proposed CNN showed comparable performance to several benchmark architectures, the differences should be interpreted cautiously due to the limited size of the independent test set. Since all models were evaluated under identical experimental conditions, the comparison provides a meaningful assessment of relative performance. The proposed CNN achieved competitive accuracy with the highest recall and F1-score while maintaining a lightweight structure, supporting its potential for automated prawn quality inspection. Future studies using larger independent datasets will enable more robust statistical comparisons and further validation of model generalizability.

### 4.6. Imaging Dependence and Practical Robustness

The excellent classification performance achieved in this study was obtained using a custom imaging system that employed backlit LED illumination to enhance contrast between the hepatopancreas and the surrounding exoskeleton. This configuration, while ideal for research purposes, creates substantially higher contrast than would be available under standard top-down lighting in commercial processing environments. It is potentially possible that classification performance would degrade if the model were deployed with conventional lighting, particularly for samples with darker pigmentation or thicker exoskeletons where the hepatopancreas is less visible through transmitted light.

Real-world processing platforms often face challenges including variable ambient lighting, debris accumulation on camera lenses, high-speed conveyor motion, and inconsistent sample positioning, all of which would likely reduce the signal-to-noise ratio and compromise classification accuracy. Without the benefit of the backlit background, the model may rely more heavily on subtle surface features rather than transmitted signatures, potentially leading to reduced sensitivity for weakly visible hepatopancreas. Furthermore, the Grad-CAM visualizations showed that the model’s attention was concentrated in the cephalothorax region under the current imaging setup; whether this localization pattern would persist under different lighting conditions remains uncertain.

To bridge the gap between laboratory performance and industrial applicability, we propose several strategies for future work: (1) training with multi-illumination datasets that include both backlit and standard top-down images; (2) employing domain adaptation techniques to align feature distributions across different imaging conditions; (3) developing robust preprocessing pipelines that can handle lower-contrast inputs; and (4) conducting systematic robustness testing with simulated or actual industrial imaging conditions. We acknowledge that the current results represent an upper-bound estimate of performance and that practical deployment will require additional validation under realistic processing conditions.

### 4.7. Justification of CNN Approach over Simple Color Thresholding

Given the distinct chromatic characteristics of the hepatopancreas and the contrast enhancement applied during preprocessing, a simple color-based thresholding method could be considered as an alternative classification strategy. In our preliminary evaluation, an HSV-based thresholding approach was conducted on the validation dataset using hue (10–25°) and saturation (>0.35) ranges corresponding to the typical orange-yellow appearance of the hepatopancreas. However, this approach achieved only 78.95% test accuracy, which was substantially lower than the CNN model. The main errors were associated with visually similar regions, pigmentation variations, specular reflections, and samples with weakly developed or partially obscured hepatopancreas.

The improved performance of the CNN model indicates that hepatopancreas classification cannot be reliably determined by color information alone. The CNN automatically integrates chromatic, spatial, and textural features, enabling discrimination between hepatopancreas-related characteristics and visually similar patterns such as shell pigmentation or illumination-related artifacts. Consistently, Grad-CAM visualization showed that the model focused primarily on biologically relevant regions around the cephalothorax [14,15], supporting that the predictions were based on meaningful anatomical features rather than isolated color differences.

Importantly, the choice of a CNN architecture was motivated not only by current performance but also by future scalability and adaptability. As the dataset expands to include additional prawn species, more diverse imaging conditions, and challenging samples such as partially occluded or immature hepatopancreas, the limitations of fixed color thresholds will become increasingly pronounced. The CNN’s hierarchical feature learning offers the flexibility to adapt to these more complex scenarios without requiring constant manual recalibration [37]. Although color thresholding is computationally simpler, its performance depends strongly on predefined color ranges and requires manual adjustment when sample characteristics change. In contrast, the CNN framework can be further refined using additional representative datasets, providing greater flexibility for future applications involving broader sample variation or more detailed quality grading tasks. Therefore, considering accuracy, feature representation capability, and interpretability, the CNN approach provides a more suitable solution for automated hepatopancreas classification.

### 4.8. Limitations and Future Research Directions

Despite these advantages, there are several limitations to this research. The dataset size, while sufficient for proof-of-concept, remains small relative to practical application which might require different batches of samples across multiple years. Although augmentation helped mitigate overfitting, additional annotated samples would likely improve performance on challenging borderline cases and enhance robustness to natural variation in pigmentation and lighting. The current binary classification framework distinguishes between “with” and “no” hepatopancreas but does not grade intermediate states, which may be desirable in more refined quality systems. Extension to a three- or four-level grading scale would require additional annotation and model modification but could provide more granular commercial information.

The distinction between effective sample diversity and unique biological samples is critical. While augmentation helped mitigate overfitting, additional biologically independent samples would likely improve performance on challenging cases and enhance robustness to natural variation. We caution readers that augmentation serves to expand feature space coverage for a given set of base samples but does not introduce new biological information. Future work must prioritize the collection of larger, independently collected datasets to validate that the learned features generalize to new biological populations.

Further, the model was trained and evaluated on images captured under controlled laboratory conditions with standardized lighting and backgrounds. Real-world deployment in processing plants may encounter variable lighting, camera exposure settings, and handling artifacts, which could degrade performance without further domain adaptation or training on more diverse data [39,40]. Although the proposed CNN achieved satisfactory performance under controlled conditions, the relatively limited dataset size remains a constraint. While data augmentation, cross-validation, and comprehensive evaluation helped improve model reliability within the current dataset, these approaches cannot fully replace validation using larger, independently collected datasets.

Future work should therefore prioritize external validation using datasets from different production batches, geographical locations, and environmental conditions to rigorously assess model robustness and generalization capability. Such validation is essential for determining the feasibility of deploying the proposed framework in practical prawn quality inspection scenarios. Additionally, given that hepatopancreas health is influenced by bacterial infections, physiological disorders, abnormal pigmentation, or dietary factors, as demonstrated by studies showing that dietary lipid levels significantly affect hepatopancreatic health and lipid metabolism in *M. rosenbergii* [41], future studies should explore the integration of spectral imaging techniques, such as near-infrared or hyperspectral imaging, which can provide additional biochemical and compositional information beyond visible appearance. Combining spectral information with deep learning may facilitate the development of more comprehensive multiclass models for simultaneous evaluation of hepatopancreas presence, quality grade, and health status.

### 4.9. Broader Implications for Crustacean Processing and Aquaculture

The feasibility of automated hepatopancreas status classification using standard RGB imaging has broader implications for crustacean processing. The methodological pipeline, including automatic cropping, enhancement, augmentation, convolutional classification, and interpretable visualization, is transferable to other quality attributes such as shell color grading, size estimation, and the detection of external disease signs. The accompanying graphical interface indicates the potential of this pipeline adopted by non-expert operators in processing facilities, thereby reducing the barrier to practical implementation. As the aquaculture industry moves toward digitalization and smart processing, such nondestructive, objective, and high-throughput systems will become increasingly needed to quality management [13,40]. The present study contributes a validated framework and an open-architecture implementation that could potentially be adapted to various crustacean species with appropriate retraining.

## 5. Conclusions

In this study, an explainable deep learning framework was developed for the binary classification of prawn images according to the presence or absence of the hepatopancreas. The proposed CNN model, trained on a carefully preprocessed and augmented dataset of 252 images, achieved a test accuracy of 97.37% and a full-sample calibrated accuracy of 97.22%. These findings indicate that, under controlled imaging conditions, a well-designed workflow incorporating automatic cropping, contrast enhancement, and data augmentation can provide accurate classification despite the relatively limited dataset size. The integration of Grad-CAM improved model interpretability by demonstrating that the classification decisions were primarily based on biologically relevant image regions. In addition, the developed graphical user interface enables rapid image analysis with visual feedback, supporting the potential application of the proposed approach in prawn quality inspection. Overall, this study provides a practical and interpretable framework for automated hepatopancreas classification and establishes a foundation for further development of image-based quality assessment systems in aquaculture.

Future work will focus on expanding the dataset to include additional prawn species, more diverse imaging conditions, and challenging samples, such as partially occluded or immature hepatopancreas, to further improve model robustness and generalization. Independent external validation using datasets collected from multiple facilities and production batches will also be necessary to evaluate the model under practical operating conditions. In addition, future studies will investigate more advanced network architectures, including transformer-based models and transfer learning strategies, as well as the continued integration of explainable artificial intelligence methods to enhance model transparency and support reliable deployment in seafood processing applications.

## Figures and Tables

**Table 1:** Statistical summary of model performance.

Dataset	Sample Number	Correct Classification	False Classification	Accuracy
Tran.	176	172	4	97.72%
Vali.	38	35	3	92.11%
Test.	38	37	1	97.37%
Full.	252	245	7	97.22%

Tran.: Training dataset; Vali.: Validation dataset; Test.: Test dataset; Full.: Full Dataset.

**Table 2:** Comprehensive performance metrics for the model performance of the test set.

Metrics	Value	95% CI (Wilson Score)
Accuracy	97.37%	86.51–99.53%
Precision (Positive)	95.83%	87.90–99.52%
Recall (Sensitivity)	100.00%	85.69–100.00%
Specificity	93.33%	86.69–97.71%
F1-Score	0.9787	88.00–99.74%
Matthews Correlation Coefficient (MCC)	0.9458	71.63–99.12%

**Table 3:** Comparison of classification performance across architectures.

Architecture	Test Accuracy (%)	Precision (%)	Recall (%)	Specificity (%)	F1-Score	MCC
Proposed CNN	97.37%	95.83%	100.00%	93.33%	0.9787	0.9458
ResNet- 18	94.74%	100.00%	91.30%	100.00%	0.9545	0.8976
EfficientNet-B0	97.37%	100.00%	95.65%	100.00%	0.9778	0.9470
MobileNetV3	94.74%	95.65%	95.65%	93.33%	0.9565	0.8899
DenseNet-121	94.74%	92.00%	100.00%	86.67%	0.9583	0.8929
VGG-16	92.11%	91.67%	95.65%	86.67%	0.9362	0.8341

MCC: Matthews Correlation Coefficient.

## Data Availability

The original contributions presented in this study are included in the article/Supplementary Materials. Further inquiries can be directed to the corresponding authors.

## Supplementary Materials

The following supporting information can be downloaded at: https://www.mdpi.com/article/10.3390/foods15162770/s1, Table S1: Key Training Hyperparameters and Model Configuration; Table S2: Threshold optimization analysis based on the Youden’s J index.

## Abbreviations

The following abbreviations are used in this manuscript:

AUC	Area under curve
CNN	Convolutional neural network
Grad-CAM	Gradient-weighted class activation mapping
GUI	Graphical user interface
LED	Light emitting diode
LSTM	Long short-term memory
ReLU	Rectified linear unit
ROC	Receiver operating characteristic
ROI	Region of interest
