# Supplementary material for: A Deep Learning-Based System for Prawn Hepatopancreas Identification Based on Image Classification with Interpretability
Source: Foods. 2026 Aug 7;15(16):2770. doi: 10.3390/foods15162770 (PMC13511993; doi:10.3390/foods15162770)
Supplement: Supplementary file 1 [file foods-15-02770-s001.zip › foods-4433425-supplementary.pdf]

Supplementary Table S1. Key Training Hyperparameters and Model Configuration

Category	Hyperparameter	Value
**Model Architecture**	Input resolution	224 × 224 × 3
	Total parameters	~2.1 M
	Convolutional blocks	4
	Convolutional layers per block	2
	Kernel size	3 × 3
	Pooling type	MaxPool (2 × 2)
	Final pooling	AdaptiveAvgPool (1 × 1)
	Fully connected layers	256–96–2
	Dropout (FC1 / FC2)	0.35 / 0.25
**Optimization**	Optimizer	AdamW
	β_1 / β_2	0.9 / 0.999
	Initial learning rate	1×10^{-3}
	Weight decay	1×10^{-4}
	Learning rate scheduler	Cosine Annealing
	Gradient clipping	1
	Loss function	Weighted Cross-Entropy
	Batch size	16
	Maximum epochs	60
	Early stopping patience	16
	Saved epoch	49
**Data Augmentation**	Random affine (rotation)	±22°
	Random affine (translation)	±6%
	Random affine (scale)	0.88 – 1.08
	Random affine (shear)	±6°
	Affine application probability	85%
	Horizontal flip probability	50%
	Vertical flip probability	25%
	Color jitter (brightness)	±0.16
	Color jitter (contrast)	±0.18
	Color jitter (saturation)	±0.18
	Color jitter (hue)	±0.025
	Random erasing probability	18%
	Random erasing scale	1.0% – 3.5%
	Random erasing aspect ratio	0.4 – 2.5
**Preprocessing**	Cache resize	384 × 384
	Model resize	224 × 224
	Normalization mean	[0.485, 0.456, 0.406]
	Normalization std	[0.229, 0.224, 0.225]
**Reproducibility**	Random seed	42
	Hardware	NVIDIA RTX 3080
	Framework	PyTorch 2.0.1 / CUDA 11.8

Supplementary Table S2. Threshold optimization analysis based on the Youden's J index

Threshold	Sensitivity	Specificity	Youden's J	F1-Score	Validation Accuracy
**0.50**	100.00%	82.22%	0.82	0.96	89.47%
**0.62**	95.65%	84.44%	0.80	0.96	89.47%
**0.68**	95.65%	88.89%	0.85	0.96	92.11%
**0.72**	91.30%	88.89%	0.80	0.94	89.47%
**0.78**	86.96%	93.33%	0.80	0.91	89.47%
**0.84**	82.61%	95.56%	0.78	0.86	86.84%
